# Comment on the risk of hepatocellular carcinoma with chronic hepatitis B infection in Sweden

**DOI:** 10.1002/hep4.2046

**Published:** 2022-07-10

**Authors:** Satender Pal Singh, Nitisha Mondia

**Affiliations:** ^1^ Department of Hepatology Institute of Liver and Biliary Sciences New Delhi India; ^2^ Department of Pediatrics Kalawati Saran Children Hospital and Lady Hardinge Medical College New Delhi India

To the editor,

We read with interest the article by Duberg et al.^[^ ^1^ ^]^ on chronic hepatitis B virus (HBV) infection and the risk of hepatocellular carcinoma (HCC) by age and country of origin in people living in Sweden. This is a population‐based register study of a national cohort of people with chronic HBV infection. The study showed that men of Asian origin who are infected with HBV should have HCC surveillance recommended at younger ages. However, certain points need to be considered.

First, this retrospective study took age and country of origin as the primary risk factors, but HBV DNA and hepatitis B e antigen (HBeAg) should have been included to know the virus status. These two important factors also play a role in HCC development in patients with cirrhosis as well as those without cirrhosis. Regrouping patients on the basis of HBeAg and level of DNA will provide more information about the hepatitis B status of the country and the infectivity.^[^ ^2^ ^]^

Second, their study should also have included comorbidities, like diabetes mellitus, obesity, and nonalcoholic fatty liver disease, which predispose patients to HCC as a single factor and increase the risk 5‐fold if combined. As these factors are associated with high insulin level and insulin resistance, insulin acts as a tumorigenic hormone.^[^ ^3^ ^]^

Last, their study included human immunodeficiency virus and hepatitis D virus coinfection; however, it should also include hepatitis C virus infection, which increases the risk of HCC manyfold.^[^ ^4^ ^]^ In addition, the study should have considered noninvasive assessment of fibrosis models, which are reported to directly correlate with HCC development as fibrosis level increases.^[^ ^5^ ^]^

## CONFLICT OF INTEREST

Nothing to report.
